# Process evaluation of Prompt Mental Health Care (PMHC): the Norwegian version of Improving Access to Psychological Therapies

**DOI:** 10.1186/s12913-020-05311-5

**Published:** 2020-05-19

**Authors:** Linn Vathne Lervik, Marit Knapstad, Otto Robert Frans Smith

**Affiliations:** 1 Division of Mental and Physical Health, Department of Health Promotion, Norwegian Institute of Public Health, Zander Kaaes gate 7, 5015 Bergen, Norway; 2 Faculty of Psychology, Department of Clinical Psychology, University of Bergen, Bergen, Norway

**Keywords:** Prompt Mental Health Care, Depression, Anxiety, Process evaluation, CBT, RCT, IAPT

## Abstract

**Background:**

Prompt Mental Health Care (PMHC) is the Norwegian adaptation of Improving Access to Psychological Therapies (IAPT). Thus far, evaluations of PMHC have mostly focused on the effectiveness, rather than on contextual and implementation processes. Therefore, the objective of this study was to do a process evaluation and examine: 1) To what extent do the services follow guidelines provided by the Norwegian Directorate of Health (NDH), 2) what the therapists experienced as important barriers and facilitators in implementing the service, and 3) client treatment satisfaction and its associations with baseline variables.

**Method:**

The present study uses data from 526 clients who received PMHC treatment in the municipalities of Sandnes and Kristiansand. The therapists completed questionnaires about each client’s course of treatment. We conducted semi-structured interviews with the therapists and analysed them using thematic analysis. Data from client questionnaires were used to report descriptive sample statistics including symptom severity and treatment satisfaction. Linear regression was adopted to examine the associations between client treatment satisfaction and baseline characteristics.

**Results:**

Several aspects of PMHC were implemented in line with the guidelines provided by NDH. Importantly, both services reached out to the intended target group, and could further be characterized as low-threshold with relatively short waiting times (median waiting time between initial contact and treatment start was 27 days, IQR 18–39), no waiting lists, and frequent use of self-referral (33.3%). From the client perspective, results indicated a high degree of treatment satisfaction (Mean = 3.93 (SD = .71, range 1–5)), and this was true across demographic characteristics and symptom severity at baseline (all *p* > .05). Most notable challenges that came forward were; the low provision of guided self-help (received by only 1.0% of clients), the lack of focus on work participation (low to some degree of focus in 70.8% among sick-listed clients), the collaboration with other services (no collaboration in 85.3% of the clients), and some aspects regarding future development of the service.

**Conclusion:**

Both sites managed to implement key aspects of PMHC in line with the guidelines, but further development of the program is warranted. Discussion of challenges and future recommendations are presented.

## Background

Psychological problems and mental disorders are among the most important causes for disease burden and reduced health worldwide, also in Norway [1]. About 20% of the adult population will experience a mental disorder each year, and anxiety and depression make up for the majority of it [2]. Anxiety and depression also are central causes for the reception of Norwegian welfare benefits, particularly for people receiving disability pension and sick leave [3–6]. According to a report by the Organization for Economic Cooperation and Development (OECD), treatment for mild to moderate mental disorders has been widely neglected, even though it makes up the largest burden of disease [7]. The median treatment gap for depression and anxiety has been estimated to be above 50% [8–10].

A country that has focused on meeting this challenge is the United Kingdom. In 2007, the National Health Service (NHS) introduced the program Improving Access to Psychological Therapies (IAPT) [11]. The objective of IAPT is to offer low threshold, free of charge, evidence-based psychological therapies for depression and anxiety disorders, in line with clinical guidelines of the National Institute for Health and Clinical Excellence (NICE) [11]. Prompt Mental Health Care (PMHC, in Norwegian; Rask Psykisk Helsehjelp) is the Norwegian adaptation of IAPT and in 2012 it was launched by the Norwegian Ministry of Health and Care Services as a pilot project in 12 municipalities [12]. Similar to IAPT, it targets people suffering from anxiety and mild to moderate depression and offers both low-intensity treatments, such as guided self-help and group-based psychoeducation, and traditional high-intensity face-to-face treatment. Although based on relatively weak research designs, initial evaluations in both countries revealed promising results [13–16], which stimulated the further implementation of the model across the respective countries. Recently, PMHC was also evaluated by means of a randomized controlled trial (RCT), in which PMHC was compared to treatment as usual (TAU) in two large Norwegian municipalities (ClinicalTrials.gov NCT03238872). Results indicated significantly higher recovery rates for the PMHC group as compared to TAU (59% vs. 32%) and large to medium between-group effect size differences in favour of PHMC for symptoms of depression and symptoms of anxiety, respectively [17].

Thus far, scientific reports from the Norwegian evaluations have mostly focused on the effectiveness of PMHC in relation to outcomes [14, 17], rather than on contextual and implementation processes [18]. However, according to Moore et al. [19], an essential part of testing complex interventions is to conduct a process evaluation as well. Moore et al. [19] argues that “*effect sizes do not provide policy makers with information on how an intervention might be replicated in their specific context, or whether trial outcomes will be reproduced*”. Based on the previous Medical Research Council (MRC) guidance [20], they propose a model for process evaluation focusing on three main, interacting components; context, implementation and factors of impact. Context is defined as “*anything external to the intervention that may act as a barrier or facilitator to its implementation or its effects*”. Implementation is divided into two main aspects; the implementation process (how delivery is achieved) and what is delivered (fidelity, dose, adaptation and reach). Factors of impact is separated into three aspects; participant’s responses to and interactions with the intervention, mediators, and consequences.

The evaluation of the first 12 PMHC pilot sites identified a number of implementation challenges that could possibly affect access to and effect of treatment, most notably non-compliance to in- and exclusion criteria, underuse of low-intensity treatments, and lack of focus on return-to-work [18]. As these were challenges identified during the early stage of development of the PMHC service, it was considered important to examine these and other potential challenges in connection with the RCT study of PMHC as well. The RCT was not only carried out at a later development stage, but also conducted at different sites, and in a different research context, which may all be associated with challenges of its own.

In the present article, we aimed to touch upon several components of process evaluation, more specifically: 1) Fidelity of recommended practices, to what extent do the PMHC services follow guidelines given by the Norwegian Directorate of Health (NDH)? 2) Context and content of the PMHC intervention; what did the therapists experience as important barriers and facilitators in implementing the PMHC service in their community? 3) Participant’s responses to and interactions with the intervention; how satisfied were PMHC clients with treatment, and to what extent were baseline characteristics associated with client satisfaction?

## Methods

The present study addressed only the PMHC treatment arm of the RCT study in the municipalities of Sandnes and Kristiansand. The data material included questionnaire and interview data from PMHC therapists and questionnaire data from PMHC clients. These data were used to examine pre-defined domains that were considered relevant for one or more of the three components of process evaluation mentioned in the introduction.

### Description of the NDH-guidelines

The NDH developed guidelines for service establishment [12]. These guidelines, together with regulations for financial support and the manual for first admission interview, formed the basis to describe and examine the implementation of PMHC in Kristiansand and Sandnes. The following section provides a brief summary of the most central characteristics of PMHC.

The main objectives of PMHC are to reduce symptoms, strengthen work ability and be an effective service at community level in offering evidence-based treatment with short waiting-times and without waiting lists. Further, it is emphasized that the service need to be low-threshold - meaning that it should be free of charge for the clients, without need for a referral, available for all in the target audience, and have personnel with competence to fulfill the purpose of the service.

It is required that the team consists of at least four full time equivalents, all with a minimum of 3 years college education in health and social studies, one clinical psychologist in at least 50% position and a part time administrative position. All therapists are required to complete an additional one-year training in cognitive behavioural therapy under the auspices of the Norwegian Association for Cognitive Therapy.

Cognitive behavioural therapy (CBT) is the therapy of choice in PMHC, and is offered both as low- (guided self-help and group-based psychoeducation) and high-intensity (face-to-face) treatment. PMHC is organized according to a so-called matched-care model, in which information from the initial assessment and client preferences is used to determine the choice of treatment. This indicates, different from the stepped-care model used in IAPT, that the client does not necessarily start at the lowest treatment level [21]. However, to maximize the possibility for treatment to all in need, the NDH recommends low-intensity treatments as first choice. The initial assessment is also used to examine whether a client is eligible for the service. Inclusion criteria are being inhabitant of the pilot site community, being ≥18 years of age, and having anxiety and/or mild to moderate symptoms of depression (formal diagnosis not provided). Clients entitled to secondary care services due to *eating disorder*, suicide risk, bipolar disorder, severe depression, *invalidating anxiety*, psychotic symptoms, severe substance abuse, personality disorder, *two or more previous treatment attempts without effect, and serious physical health problem as prime problem disorder* are generally excluded from PMHC and are referred elsewhere. The italicized exclusion criteria are assumed to be less frequently observed in the context of PMHC and are therefore not mentioned in the NDH-guidelines, but were added to the RCT-protocol for the sake of completeness.

During treatment, the guidelines emphasize the need for regularly evaluation of treatment progress through symptom monitoring, the importance of collaboration with other services (in particular general practitioners (GP)), and to ensure user involvement of clients and dependents. Municipalities and Oslo boroughs can apply for funding from the NDH. If granted, PMHC is established through a funding scheme lasting up to 4 years. After this period, local government funding is required for the continuation of the service.

### Site description

The sites in the present study are located in the southern part of Norway, Kristiansand on the South and Sandnes on the West coast. Based on population figures from 2016, Kristiansand had in total 88,447 and Sandnes 74,820 residents [22]. Relative to the national average of inhabitants having immigrant background (16.3%), higher education (36.0%) and unemployment (2.3%), the sites were similar to each other and slightly above the national average on these characteristics. However, in comparison to the proportion of people on permanent disability pension (9.0%) and work assessment allowance (AAP, 4.6%) in Norway, the figures for Sandnes were just below (7.2% resp., 3.4%) and Kristiansand (10.0% resp., 5.1%) just above the national average in the period 2014–2016 [22].

### Procedures

All clients contacting PMHC were offered a first assessment session. During this session, the therapist explained the rationale for the study, provided information about the PMHC treatment, and assessed if the treatment was suitable for the client. Clients were asked to complete an electronic baseline questionnaire and were subsequently randomized to PMHC or treatment as usual (TAU). More information about the in−/exclusion criteria, randomization procedure and TAU condition can be found elsewhere [17].

Clients allocated to the PMHC treatment were asked to complete questionnaires before each session, at post-treatment, and at 6-, 12-, 24-, and 36-month follow-up. For all PMHC-clients, the therapists were asked to complete a questionnaire regarding the treatment process for each client by the end of the treatment period.

The study was approved by the Regional Ethics Committee for Western Norway (REK-vest no. 2015/885). All participants provided written informed consent.

### Participants

Between Nov 9, 2015, and Aug 31, 2017, 1189 clients were assessed for eligibility. Of these, 353 did not meet the inclusion criteria, 35 declined treatment and 26 declined trial participation. A total of 774 clients participated in the study, of which 526 were assigned to the PMHC group.

### Measures

#### Therapist variables

Two questionnaires were given to the therapist; one at the beginning of the trial in which therapist characteristics were assessed (e.g. professional background, primary therapeutic orientation, familiarity to CBT, experience with CBT and treatment in general). In addition, the therapists completed a process form for each client after the end of treatment in which they reported client and session characteristics such as total amount of treatment sessions, type of treatment provided and reason for termination. For 510 of the included 526 clients (97.0%), the process form was completed, although completion rates were lower for a number of process form variables, in particular the waiting time variables (up to 20% missing) and the treatment type variable (≈10% missing).

#### Client variables

Symptoms of depression were measured by means of the Patient Health Questionnaire (PHQ-9). This is a nine-item scale with four response categories ranging from 0 (“not at all”) to 3 (“nearly every day”) [23, 24]. The PHQ-9 has been shown to have good psychometric properties [23] and in our sample the Cronbach’s alpha for the instrument was 0.80. A sum score was created, ranging from 0 to 27.

To measure symptoms of anxiety, we used the Generalized Anxiety Disorder Assessment (GAD-7). This is a seven-item scale with response categories ranging from 0 (“not at all”) to 3 (“nearly every day”) [24, 25]. GAD has been found to have good reliability and validity for measuring generalized anxiety disorder [25] and to have satisfactory sensitivity and specificity for generalized anxiety as well as other anxiety disorders [9]. In our sample, the Cronbach’s alpha for the instrument was 0.83. A sum score was created, ranging from 0 to 21.

The client satisfaction scale used in the present study was partly based on the patient experience questionnaire that is available on the IAPT website (item 1–5) [26], and partly based on a questionnaire that was used in a Norwegian multicentre study (item 6–10) [27]. The scale uses a 5-point Likert scale, ranging from “to a very small degree” to “to a very large degree”. The scale was completed at post-treatment by 62% (*n* = 326) of the PMHC participants. For the computation of the sum score, item 9 was recoded in the same direction as the other items such that a higher score indicated a higher degree of client satisfaction. In our sample, the Cronbach’s alpha for the instrument was .91.

### Interview data

Semi-structured interviews were conducted with three team-members in Sandnes and four team-members in Kristiansand to obtain information about the implementation of PMHC at these two sites (see Additional file 1 for the entire interview guide). The interview duration was approximately 60 min, conducted at the PMHC site and digitally recorded. The recordings were stored at a secure server at the Norwegian Institute of Public Health.

The interview guide for the therapist interviews was based on the previous Norwegian evaluation of PMHC by Smith, Knapstad and Alves 2016, on the English evaluation of IAPT by Parry et al. 2011 and on the NDH guidelines. Questions specifically related to the RCT were also added because it was anticipated that study participation may have influenced service routines and therapist behavior, and could as such have served as a potential facilitator/barrier for the implementation of PMHC.

### Analyses

Two important perspectives within implementation theory were adopted to examine the interview data [28]. The top down perspective was used to examine to what extent the guidelines as formulated by the NDH have been followed by the two PMHC sites. The bottom up perspective was used to focus on how implementation processes have contributed to solve the problem of limited access to evidence-based treatment for people with anxiety and mild-to-moderate depression. The latter puts a stronger emphasis on the roles, views, actions and experiences of the locally involved. The reason for using both approaches was to obtain a more comprehensive understanding of the extent to which the implementation was successful, as well as obtaining more information about the possible impact of deviations from the NDH guidelines on the effectiveness of the intervention.

Thematic analysis [29] with a framework approach [30] was used to identify themes from the interviews within predefined domains based on the guidelines from NDH and the previous thematic analysis from the first Norwegian evaluation. See Table 1 for the full list of predefined domains. Thematic analysis is a method for identifying, analysing and reporting patterns (themes) within data [29], and often contains six steps: 1) Familiarize with the data, 2) assign preliminary codes to the data in order to describe the content, 3) search for patterns or themes in the codes across the different interviews, 4) review themes, 5) define and name themes, and 6) produce the report. Due to the relatively focused and confirmatory nature of the interview questions and the predefined domains, notes taken during and directly after the interviews were used as the primary source to identify themes. The interviewer/analyst (LVL) transferred all interview notes into an excel spreadsheet marking each interview text with a different colour to get a better overview. If the excel notes were considered unclear and/or incomplete, the analyst (LVL) listened to the recordings again, in order to make sure that all relevant information was included.

**Table 1:** Predefined domains and themes from the therapists interviews

Domains	Themes
*Organisation and establishment of a new service*	• Motivated employees • Pleased with skills training • All team-members contributed to the establishment • Challenge to promote PMHC and guided self-help • Site stability
*Target group of PMHC*	• Compliance with criteria • A wide range of clients included from all classes of society
*Use of low-intensity treatment*	• Guided self-help to a limited extent, perceived as more high-intensity treatment • Good experiences with group-based psychoeducation
*Focus on work participation*	• Overall low degree of work-focus • No routine for work-focussed treatment
*Collaboration with other services*	• Low frequency of collaboration • Mainly good experiences
*User involvement*	• Important part of the CBT methodology • Various degree of user involvement depending on level of treatment
*Client satisfaction*	• Majority of clients satisfied with treatment
*Continuation of the service through local government funding*	• Continuation with a slightly different organisational structure • Maintain the target group and competence of the therapists
*Participation in an RCT*	• No major differences in daily routines • More true to the model and the target group
*Suggestions for future changes of PMHC*	• Several suggestions, no common theme

Descriptive statistics were computed using Stata version 15.0. Means and standard deviations (SD) were reported for continuous variables; medians and interquartile ranges (IQR) for single items with Likert scales and count variables, and frequencies and percentages for other categorical variables. Chi-square tests were used for associations between categorical variables. Mann-Whitney U test was used to compare medians between two independent groups. Linear regression was used to examine baseline predictors of client satisfaction. The following baseline variables were included: sex, age, educational level, job status, immigration background, site, PHQ-score, and GAD-score.

## Results

The results below are presented in accordance with the structure of the research aims 1–3. Both quantitative and qualitative results are presented for each section with the exception of the section about low-threshold service performance characteristics, which was solely based on quantitative data.

### Fidelity of recommended pratices; to what extent do the PMHC services follow guidelines given by the Norwegian Directorate of Health (NDH)?

#### Low-threshold service performance characteristics

A large proportion of referrals were pure self-referrals (33.3%, *n* = 167), and this was most pronounced in Kristiansand (Table 2). Moreover, 50.0% of the clients were recommended by their GP to make contact with the service. Only 9.8% (*n* = 49) of the clients was referred directly by their GP (Table 2). It should be noted that referral type was not associated with any of the included demographic variables (age, gender, educational level, marital status, employment status, immigrant background). As such, the possibility of self-referral did not contribute to improve access for typically underrepresented groups. See sub-section “Target group of PMHC” for more details about the underrepresented groups.

**Table 2:** Descriptive statistics by site

	Total	Kristiansand	Sandnes	Test statistic
PHQ-9 Baseline, % (n)				χ^2^(4) = 2.18
None (0–4)	2.5 (13)	1.8 (4)	2.9 (9)	
Mild (5–9)	18.3 (96)	17.0 (37)	19.2 (59)	
Moderate (10–14)	33.3 (175)	32.6 (71)	33.8 (104)	
Moderately severe (15–19)	31.2 (164)	31.7 (69)	30.8 (95)	
Severe (> 19)	14.8 (78)	17.0 (37)	13.3 (41)	
GAD-7 Baseline, % (n)				χ^2^(3) = 6.65
None (0–4)	8.0 (42)	7.3 (16)	8.4 (26)	
Mild (5–9)	27.0 (142)	23.4 (51)	29.5 (91)	
Moderate (10–14)	39.0 (205)	37.6 (82)	39.9 (123)	
Severe (> 14)	26.1 (137)	31.7 (69)	22.1 (68)	
Work status baseline, % (n)				χ^2^(2) = 10.88**
Work no support	37.8 (199)	39.4 (86)	36.7 (113)	
Work with support	37.3 (196)	29.8 (65)	42.5 (131)	
Not at work	24.9 (131)	30.7 (67)	20.8 (64)	
Waiting times, median (IQR)				
Days between initial contact and assessment	12 (7–17)	12 (7–17)	12 (7–17)	U = 28611
Days between assessment and first treatment session	13 (8–23)	15 (9–28.5)	13 (7–22)	U = 19309*
Days between initial contact and first treatment session	27 (18–39)	28 (18.3–42.8)	27 (18–38)	U = 21759
Therapy duration, median (IQR)				
Number of sessions	4 (4–9)	4 (3–6)	5 (4–10)	U = 38350***
Number of weeks	9.4 (4.9–21.1)	7.4 (4–16)	10.7 (4.7–24.7)	U = 40080***
Treatment form, % (n)				χ^2^(3) = 9.70**
Guided self-help	1.0 (5)	2.1 (4)	.3 (1)	
Group-based psychoeducation	36.5 (175)	40.8 (78)	33.7 (97)	
Individual CBT	29.4 (141)	30.9 (59)	28.5 (82)	
Mixed	33.0 (158)	26.2 (50)	37.5 (108)	
Referral, % (n)				χ^2^(2) = 25.75***
GP	9.8 (49)	5.8 (12)	12.5 (37)	
Self, recommended by GP	50.0 (251)	44.7 (92)	53.7 (159)	
Self	33.3 (167)	45.1 (93)	25.0 (74)	
Others	7.0 (35)	4.4 (9)	8.8 (26)	
Work-focus in treatment, median (IQR)				
Full sample	3 (2–3)	2 (1–3)	3 (2–4)	U = 40752***
Sick-listed at baseline (*n* = 187)	3 (2–4)	3 (1.8–3)	3 (3–4)	U = 4624***
Collaboration with external parties, % (n)†				
None	85.3 (435)	91.5 (194)	80.9 (241)	χ^2^(1) = 11.17***
GP	8.8 (45)	2.4 (5)	13.4 (40)	χ^2^(1) = 18.85***
Secondary care services	0.8 (4)	0 (0)	.8 (4)	χ^2^(1) = 2.89
Work and social services	2.8 (14)	.9 (2)	4.0 (12)	χ^2^(1) = 4.41*
Work place	0.8 (4)	.5 (1)	1.0 (3)	χ^2^(1) = .46
Family	2.2 (11)	.5 (1)	3.4 (10)	χ^2^(1) = 4.88*
Others	3.9 (20)	3.3 (7)	4.4 (13)	χ^2^(1) = .37

*p < .05, **p < .01, ***p < .001, ^†^treated as independent binary variables

The median waiting time between initial contact and assessment was 12 days (IQR 7–17) and between initial contact and first treatment session the median was 27 days (IQR 18–39). The median number of sessions that a client received was 4 (IQR 4–9) and the median length of treatment was 9.4 weeks (IQR 4.9–21.1, see also Table 2).

#### Organisation and establishment of a new service

Each PMHC team started with four full-time equivalents (four therapists in Sandnes, seven therapists in Kristiansand). Each site had a psychologist who carried the professional responsibility. All therapists had higher education and nearly half of the therapists had attained a master degree (45%, *n* = 5). Most of them had over 5 years of experience with face-to-face treatment (72.7%, *n* = 8).

Throughout the project period, Sandnes had a clearly defined manager working fulltime in the project with both client and staff responsibility, whereas the team in Kristiansand was less stable in this regard and changed manager several times. Moreover, three therapists from Kristiansand, including the psychologist, quitted during the project period and were not replaced. As a result, Kristiansand was understaffed for a relatively long period of time. Both sites experienced challenges related to sick- and maternity leaves.

#### Target group of PMHC

Most participants reported symptom levels that were in line with the intended PMHC target group (the mild to moderate range, see Table 2). The percentage of participants under clinical cutoff for both symptoms of depression (PHQ < 10) and anxiety (GAD< 8) was 12.0% (*n* = 63), whereas the percentage of participants with severe depression was 14.8% (*n* = 78).

The symptom scores gave an indication on the severity of anxiety and depression, but the clinical presentation during the initial assessment also played a part in deciding whether a client was within the target group. Some deviations from the target group regarding symptoms level, as we reported here, are therefore to be expected. As displayed in Table 3, a wide range of clients from all classes of society was included in the study, which was in line with the therapists’ own experience, as reported in the interviews (Table 1). However, when comparing the sample statistics with the municipality statistics, a number of deviations should be mentioned. Both for Kristiansand and Sandnes, the PMHC sample appeared to be underrepresented by men, middle to old age individuals (45–64 years), individuals with lower education, people with disability pension, and likely individuals with immigrant background (Table 3).

**Table 3:** Sociodemographic characteristics by pilot site and by municipality, % (n)

	PMHC Total	PMHC Kristiansand	Kristiansand Municipality^a^	PMHC Sandnes	Sandnes Municipality^a^
Gender					
Women	65.2 (343)	59.2 (129)	50.1 (44307)	69.5 (214)	48.9 (36581)
Men	34.8 (183)	40.8 (89)	49.9 (44140)	30.5 (94)	51.1 (38239)
Age group					
≤ 19 years	4.4 (23)	3.2 (7)	−^b^	5.2 (16)	−^b^
20–24 years	18.1 (95)	22.9 (50)	7.6 (6740)	14.6 (45)	6.5 (4871)
25–44 years	56.3 (296)	52.8 (115)	27.8 (24554)	58.8 (181)	30.9 (23133)
45–64 years	19.4 (102)	18.8 (41)	24.5 (21682)	19.8 (61)	23.3 (17423)
≥ 65 years	1.9 (10)	2.3 (5)	−^b^	1.6 (5)	−^b^
Education level of full sample					
Primary School	9.6 (50)	7.4 (16)	−^b^	11.2 (34)	−^b^
Secondary School	46.1 (240)	46.5 (101)	−^b^	45.7 (139)	−^b^
Higher education	44.3 (231)	46.1 (100)	−^b^	43.1 (131)	−^b^
Education level for ≥25 years					
Primary School	7.7 (31)	5.0 (8)	18.5	9.4 (23)	20.6
Secondary School	38.4 (155)	36.9 (59)	41.7	39.3 (96)	42.5
Higher education	54.0 (218)	58.1 (93)	39.8	51.2 (125)	36.9
Immigrant background	12.0 (63)	12.8 (28)	17.5^c^	11.4 (35)	21.1^c^
Disability pension					
full sample	2.3 (12)	2.8 (6)	−^b^	2.0 (6)	−^b^
age-group 18–66	2.3 (12)	2.8 (6)	9.5^d^	2.0 (6)	6.4 ^d^
Unemployment					
full sample	14.3 (75)	16.1 (35)	3.2^e^	13.0 (40)	4.1^e^
age-group 30–74	13.7 (42)	17.2 (20)	3.0	10.8 (22)	4.0
AAP					
full sample	7.0 (37)	9.2 (20)		5.5 (17)	
age-group 18–66	7.1 (37)	9.3 (20)	5.1	5.6 (17)	3.4

^a^data collected from www.ssb.no, 2016. ^b^for these samples/categories, we do not have comparable numbers at the municipality level. ^c^municipality statistic covers the entire age 0–99, and is as such not fully comparable with our statistic. ^d^proportion of people on disability pension,2014–2016. ^e^age 15–74

#### Use of low-intensity treatment

According to the quantitative therapist-data, low-intensity treatment was primarily provided in the form of group-based psychoeducation (36.5%, *n* = 175), whereas only 1.0% (*n* = 5) of the clients received guided self-help. A mixture of low- and high-intensity treatment forms was used by 33.0% (*n* = 158) of the clients. In total, 70.6% (*n* = 338) of the clients received some form of low-intensity treatment. This was in contrast with the evaluation of the first twelve pilots, where mere face-to-face treatment was used by about two-thirds of all the clients, and some form of low-intensity treatment by the remaining one-third [31]. The extent to which low- and high-intensity treatment was used were similar in both sites with the exception of clients who received a combination of low- and high-intensity treatment, which was larger inn Sandnes (37.5%, *n* = 108) as compared to Kristiansand (26.2%, *n* = 50), see also Table 2.

#### Focus on work participation

One of the key outcomes for PMHC according to the guidelines by NDH is to strengthen work ability by having an integrated work-focus in treatment. Therapists were therefore asked to rate the overall degree of work-focus in treatment on a scale from 1 (very low) to 5 (very high) for each client by the end of treatment. The Median work-focus in treatment was 3 (IQR = 2–3), which suggested that the majority of therapists reported low to some degree of work-focus in treatment, but relatively few reported high-to-very high degree of work-focus in treatment (20.7%, *n* = 103). Focus on work participation was generally higher in Sandes as compared to Kristiansand (Table 2). For sick listed clients (*n* = 178), for whom the integrated work-focus in treatment is likely to be most relevant, the median score was also 3 (IQR = 2–4), although the percentage of therapists reporting high-to-very high integrated work-focus was somewhat higher (29.2%, *n* = 52). Overall, these results indicated that the level of reported integrated work-focus in treatment was not on par with the NDH guidelines. These results are also in accordance with results from the interviews, in which the therapists reported an overall low degree of work-focus in PMHC treatment. However, the perceived lack of work-focus was more pronounced in Kristiansand than in Sandnes in line with the quantitative data. Information from the interviews suggested that therapists from Sandnes were more inclined to have work-focus in face-to-face treatments, in particular for clients on sick-leave.

#### Collaboration with other services

The guidelines by NDH emphasized collaboration with other services, also during treatment, and particularly with GP’s. Contacts with external parties in relation to individual clients were relatively scarce according to the therapist questionnaires. The therapists reported having no such contact in the majority of cases (85.3%, *n* = 435). In 8.8% of the cases (*n* = 45), contact with GPs were reported. Contact with other agencies, such as social services, work place and secondary services were rare. There was generally more collaboration with external services in Sandnes as compared to Kristiansand (Table 2). Reasons given for collaboration were to clarify the client’s condition, to discuss medication, sick leave, and/or return to work, to give information about the PMHC treatment, to prevent relapse, to provide support, to refer clients to other services, and to consider the care situation for children. Although these client-level data provide some indication on the degree of collaboration with other services, collaboration was also established at a more general level through planned meetings, such as the ones mentioned in the earlier paragraph during the establishment phase of PMHC. Information from the therapist interviews also pointed to that there was relatively little collaboration with other services, in particular at the client level and with services other than GP’s. During the times they did collaborate, they expressed mainly good experiences. Good collaboration was reported with both GP’s and other primary care services. Secondary care services was reported to be more involved in the beginning. Eventually, they received few referrals from them. Some therapists mentioned that they could have collaborated more, especially with work and social services.

#### User involvement

According to the interviews, all therapist considered user involvement at some level. Some therapists thought of it as a fundamental part of the CBT methodology in which the overarching goal is to help clients becoming their own therapists. In this sense, clients need to be involved in order to benefit from treatment.

### Context and content of the PMHC intervention; what did the therapists experience as important barriers and facilitators in implementing the PMHC service in their community?

#### Organisation and establishment of a new service

When asked to what extent CBT appealed to them personally, the median therapist score was 5 (IQR = 5–5) on a scale from 1 (not at all) to 5 (very much). In addition, all the interviewed therapists reported high degree of satisfaction with the one-year mandatory training by the Norwegian Association for Cognitive Therapy.

Other related themes from the interviews were that they all expressed high degree of motivation for the project. One therapist described the start-up process as “being in love with the project”. They also experienced being part of establishing PMHC in their municipality. Topics mentioned were in terms of developing various routines, such as designing first session assessment and group-based psychoeducation. Both sites spent time in the beginning to promote themselves in the local community through meetings with possible collaborators and advertising. Some therapists reported that it was a challenge to convince partners from other services that low-intensity approaches such as guided self-help can be effective and sufficient to treat anxiety and depression. Scepticism was in particular about the use of telephone contact in treatment of clients. It was also mentioned, that they had to deal with questions regarding the necessity of providing a service for this relatively resourceful target group.

Therapists reported some degree of turmoil from both sites, although this seemed more pronounced in Kristiansand. Therapists from the latter site mentioned lack of hierarchical structure and associated time consuming decision-making processes as a source of discontent. The majority of therapists expressed that they did not believe team instability had influenced the quality of the service because they handled things along the way. However, some of them expressed uncertainty in this regard. To some degree, team instability might have had an impact on clients receiving group-based psychoeducation with only one therapist instead of two, or in individual treatments because of delays or premature ending of the therapeutic relationship. Other possible impacts noted were in terms of less supervision and less promotion of the service in hectic periods. One obvious impact related to staff problems in Kristiansand was the difference in number of clients seen during the project period, which was a factor 1.41 higher in Sandnes.

#### Use of low-intensity treatment

Therapists at both sites stated that they provided guided self-help to a limited extent until they started with an internet-based program,” www.assistertselvhjelp.no” [“assisted- self-help”], towards the end of the data collection period. According to the therapists, reasons for the limited use of guided self-help were clients wanting face-to-face therapy and the experience of guided self-help as more high-intensity than the group-based psychoeducation. The latter was mainly because they did not have a clear guided self-help manual. However, some therapists also mentioned that the distinction between group-based psychoeducation and guided self-help was less clear since they also provided information about self-help literature in the groups. In addition, all therapists expressed that they recommended clients to start with the group-based psychoeducation as it was perceived as effective, it helped to reach more of the target population, and it prevented waiting lists. It should be noted that the team members themselves developed the group-based psychoeducation in both Sandnes and Kristiansand. They used other PMHC sites’ group-based psychoeducation courses as inspiration, together with literature from the Norwegian Association for Cognitive Therapy and their own competence. According to the therapists in Kristiansand, they were particularly inspired by the course in Sandnes.

#### Focus on work participation

All the therapists indicated that they did not have a routine for integrated work-focus in treatment, except for asking about work status during first assessment. Other aspects were mostly handled by therapists individually in consultation with clients.

Examples of ways to address work-focus in treatment mentioned by the therapists were that they participated in meetings and talked to employers if the client wanted to, and that they had work as a topic throughout treatment for sick-listed clients. One therapist mentioned that they had more work-focus in the early development stage of group-based psychoeducation but that it later changed to focus on activity. It was not clear from the interview what caused this change. Nonetheless, this therapist believed that there was a potential to include more work-focus in the group-based psychoeducation. In contrast, another therapist stated that work focus in treatment was not necessary for this particular target group because they were relatively self-sufficient in this regard.

#### Collaboration with other services

Presented facilitating factors for collaboration were the use of e-messages to GP’s, keeping GP’s informed about the service by participating in meetings, and having clients that participated simultaneously on courses regarding lifestyle changes. Described barriers for collaboration were lack of referrals, particularly therapists mentioned that some GP’s did not want to refer clients when they were part of the RCT study due to the possibility of receiving the client back as part of the treatment as usual group. It was also mentioned that collaboration was established only if the client wanted it.

#### User involvement

One method that PMHC Kristiansand adopted to secure user involvement was Feedback Informed Treatment. They used this systematically with all clients both if they were part of the group-based psychoeducation and in individual treatment sessions. PMHC Sandnes mentioned another example of user involvement, in which participants were allowed to decide what topics to cover in group-based psychoeducation. This form of user involvement was primarily used during the early phase of the project. Later, the staff decided on a fixed course structure based on previous demand for topics in order to increase work efficiency.

#### Continuation of the service through local government funding

Both sites are continuing their services, however, in a slightly different organisational structure than originally, according to the therapist interviews. PMHC Kristiansand merged with two other municipalities, but there was some controversy as to how the service should be organized. They are now part of the mental health, alcohol and drug unit and some would like them to be part of the municipality’s unit for health promotion and lifestyle changes. A supporter of the current situation stated that this may have a positive influence on the more severe cases, while others expressed they wanted to be associated with less severe cases and life management. It is currently not clear which consequences this may have for the general functioning of the service.

PMHC Sandnes has become part of their municipality’s unit that offers a variety of services mainly to more severe cases. Therapists in Sandnes has expressed concerns about the PMHC-service due to the fact that anyone working at the unit should provide PMHC tasks without having the training in CBT. Some are afraid that changing the target group and using untrained personnel could affect the quality and effectiveness of the provided treatment.

Across sites, therapists mentioned a desire to maintain the target group and to quality assure the therapists e.g. through the education from The Norwegian Association for Cognitive therapy. Additionally, both sites are continuing to develop alternative modes of treatment, such as psychoeducational courses for specific groups.

#### Participation in an RCT

Overall, the therapists reported that having been part of an RCT did not affect their workday that much. One change in practice due to RCT-participation was that a potential client always had to meet for an assessment interview, while before, this was often conducted on telephone. Another change was the increase of forms to fill in. Positive remarks were that it was educational, it made them more true to the model and the target group, the capacity was less of a challenge because of the 30% randomisation to the TAU-group, and that it gave them credibility. More critical remarks were the use of the TAU group, in which one therapist experienced it as ethically challenging when a client was randomized to this group knowing that the person most likely would not get alternative treatment otherwise. One therapist expressed to never get used to recording sessions.

#### Suggestions for future changes of PMHC

The therapists mentioned a number of topics during the interviews; additional collaboration with secondary care services, inclusion of other treatment approaches such as body-oriented or emotion-focused therapy, development of group-based psychoeducational courses for specific groups (i.e. burnout), drop-in for panic disorder, more use of internet treatment, integrate collaboration with job specialists, requirements for regular supervision by experienced CBT therapist and continuing to use the questionnaires from the research period.

### Participant’s responses to and interactions with the intervention; how satisfied were PMHC clients with treatment, and to what extent were baseline characteristics associated with client satisfaction?

**Fig. 1:**
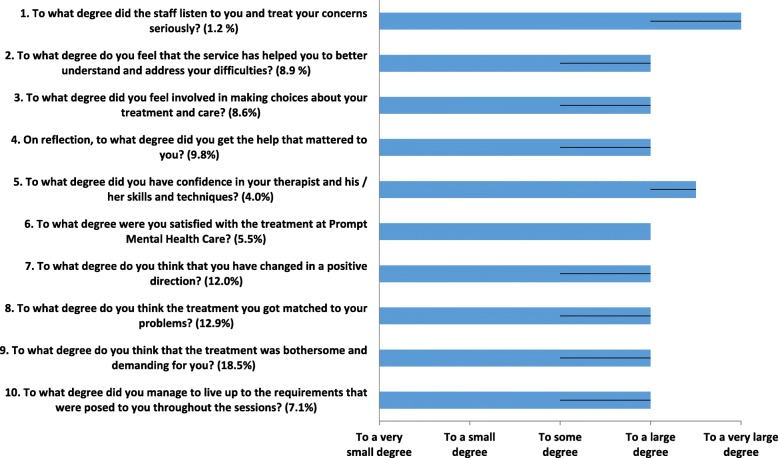
Median client satisfaction based on a random sample of clients who completed the post-treatment assessment (*n* = 326). In parentheses is the percentage of clients who answered “to a very small degree” or “to a small degree”. Interquartile ranges are denoted by the black lines

According to the therapist interviews the majority of clients were satisfied with treatment. This is in line with quantitative results from the clients. Figure 1 provides an overview of reported client satisfaction with various aspects of the PHMC service. Participants indicated that they were satisfied with the treatment (question 6). Specifically, clients were satisfied with how PHMC contributed to improving their mental health (questions 2, 4, 7 and 8), with the PHMC therapist (questions 1 and 5), and to what extent they were involved in the treatment itself (Question 3). The clients also indicated that the treatment required a fair amount of self-effort (questions 9 and 10). Overall, these results suggest a high degree of client satisfaction with PHMC (Mean score across items was 3.93 (SD = .71). As also shown in Fig. 1, there were very few clients dissatisfied with PHMC, and only a low percentage of clients selected categories indicating that they were satisfied “to a very small degree” or “to a small degree”. Baseline variables sex, age, educational level, job status, immigration background, site, PHQ-score, and GAD-score did not predict overall treatment satisfaction at the *p* < .05 level in unadjusted linear regression analyses.

## Discussion

### Main findings

Overall, the findings from this study suggest that both sites managed to implement key aspects of PMHC in line with the guidelines provided by NDH. Importantly, the PMHC service in both sites reached out to the intended target group, and could further be characterized as low-threshold with short waiting times, no waiting lists, and frequent use of self-referral. The NDH has suggested that a crucial way to avoid waiting lists in the presence of limited resources is the use of low-intensity treatment forms, which according to the results was received by more than two-thirds of the clients. PMHC Kristiansand struggled to maintain staffing at the desired level for longer periods of time as they were not able to replace team members that quitted. This resulted in PMHC Kristiansand being able to treat considerable less clients than PMHC Sandnes during the project period.

Important facilitators for implementing the service that were identified through the therapist interviews were; highly motivated employees, the training by the Norwegian Association for Cognitive Therapy, site stability and in particular a clearly defined manager working full time in the project with both client and staff responsibility (Sandnes), use of e-message to GP’s, Feedback Informed Treatment to secure user involvement, majority of clients satisfied with treatment and being part of an RCT which made them more true to the model and the target group**.** Important barriers were; to promote PMHC and guided self-help, site instability (Kristiansand), lack of high-quality material for guided self-help, low degree of integrated work-focus in treatment, low frequency of collaboration, and the transition from a centrally to a locally funded service.

From the client perspective, results indicated a high degree of treatment satisfaction at each site, and this was true across demographic characteristics and symptom severity at baseline.

The results summarized above point to a number of overarching challenges, most notably regarding the provision of guided self-help, the low degree of focus on work participation, the collaboration with other services, and the future development of the service. Each of these challenges is discussed in more detail below.

### Use of low-intensity treatment

Frequent use of evidence-based forms of low-intensity treatment (group-based psychoeducation and guided self-help) are vital to meet the primary aim of IAPT and PMHC alike, namely to improve the access to psychological therapies. An evaluation carried out during the first phase of PMHC in 12 pilot sites clearly indicated a struggle to utilize low-intensity treatment forms [18]. This was mainly due to lack of training and availability of material [18]. In Kristiansand and Sandnes, sites that initiated the service at a later stage, a marked increase in the use of low-intensity treatment forms was observed, in particular group-based psychoeducation. Guided self-help was still severely underutilized during the data collection period of the RCT, and lack of high-quality self-help material was considered a key reason for this. Obviously, this poses a barrier for a viable and cost-effective implementation of PMHC and making high-quality self-help alternatives available to all PMHC sites should clearly be prioritized. It is likely that many more clients could have received treatment if guided self-help would have been implemented properly [13]. It is possible that the failed implementation of guided self-help can partly be attributed to the therapists, but it’s more conceivable that structural factors on a higher level have played a more decisive role, such as lack of binding conditions regarding the use of low-intensity treatment, too little training, and the already mentioned lack of high-quality material. It should be noted though that both Kristiansand and Sandnes increasingly used this level of treatment towards the end of the data-collection period by implementing an internet-based self-help program. The use of internet-based self-help programs is particularly relevant for countries like Norway, with many people living in low population density areas for whom access to regular services is limited. Although more research on the subject is needed, there is increasing amount of empirical evidence supporting that these programs, are effective [32–34], and pose as such a viable alternative to traditional face-to-face treatment. As such, IAPT has also expressed the ambition to increase the use of internet-based treatment programs in their services [35]. The discrepancy between the NDH recommending the use of low-intensity treatment forms on one hand and not facilitating its implementation on the other hand could be considered as an illustration of how theory and practice are not always on par with each other, and could serve as an important lesson for other countries that want to adapt the IAPT model.

### Focus on work participation and collaboration with other services

In this study, we found an overall low degree of focus on work participation in treatments at both PMHC sites, although this was more pronounced in Kristiansand. This is in contrast to the objective of the PMHC guidelines, which emphasizes an integrated work-focus as a means to improve work capacity. This discrepancy was present in evaluation of the first 12 sites as well, where notably an even lower degree of work-focus was indicated by clients than therapists [18]. Taking the uncertain effect of PMHC on work participation into account [17], it is undoubtedly relevant to consider increased integrated work-focus in therapy as a factor that may improve this outcome.

One important barrier identified from the therapist interviews for the limited focus on work participation was that they did not have a clear procedure for how to integrate this into treatment, despite having received training in work-focused CBT. Instead, focus on work participation was integrated in a more ad hoc fashion by each therapist individually. This may partly be due to the fact that a formal manual for work-focused CBT was not available prior to autumn 2018, which is after the data collection period. According to a recent systematic review, it is highly important to use specific work-focused CBT programs for mental health conditions to optimize the effect on return to work [36]. These programs typically highlight developing work-related goals, addressing work in every session and throughout the therapy together with provision of a checklist for work-focused CBT and a schema to assess clients’ work situation, all examples of aspects that likely should be emphasized more if aiming for increased work participation. Collaboration is also recommended, mainly with GP’s, employers and work and social services, notably, tailored to the needs of each client. Our results indicated a very low degree of collaboration with other services. One may, thus, speculate whether structural implementation of work-focused CBT, would in fact increase both work-focus and collaboration with other services, and presumably also work participation as an outcome.

An alternative model to improve work outcomes was mentioned in a report issued by the English government, and recommended to pilot an intervention combining the Individual Placement and Support (IPS) model with IAPT to increase employment status for people with common mental health problems [37]. IPS is an evidence-based model for work participation originally developed for severe mental illnesses in the United States [38], increasingly implemented also for other conditions [39–42]. The IPS model is characterized by integrating job specialists in treatment teams, where obtaining and sustaining work is part of the treatment objective [43]. The IPS in IAPT pilot model was tested in four IAPT sites in England in 2014. The evaluation indicated positive feedback from all stakeholders, but also emphasized important obstacles with regard to implementation and organisation, especially barriers regarding effective collaboration [44]. The latter is repeatedly reported to be a challenge in return to work interventions for this group across several countries [45]. A second pilot phase has been initiated. These experiences may be highly relevant to consider for PMHC and similar models as well.

### Future development of PMHC

After the initial 4-year pilot period funded by the central government, both sites are now supported by the local municipalities. This change in funding scheme was not just a formality, but had important implications for the organization of the service. From the therapist interviews, it became clear that both PMHC sites had continued their services in a somewhat different organisational format. A related theme was therapists’ concern regarding alteration of the target group and the competence of the therapists due to the organisational changes. Additionally, both sites are continuing to develop alternative modes of treatment, such as psychoeducational courses for specific groups. It cannot be precluded that these developments following the transition from central to local funding may pose a threat to the effectiveness of the service, since these novel aspects have not been evaluated.

Interestingly, from the therapist interviews being part of an RCT was not considered a barrier for the implementation process. Instead, most therapists reported that it helped them to be more true to the treatment model. In light of the abovementioned issues about changes in the service, this is also an aspect to consider for further implementation. When monitoring through the RCT is over, will the therapists become less strict with inclusion criteria and more lenient in terms of treatment provided?

A crucial difference between the evaluations of PMHC and IAPT is the unique built in session-to-session continuous monitoring of the IAPT services. Clinical outcome of the clients are systematically collected and some statistics are also made public available [35]. This feature has allowed for ongoing evaluation and provided increased transparency on the effectiveness of IAPT. Monitoring has also contributed greatly to research on various aspects of the service that in turn has provided valuable knowledge and recommendations for improvements. A recently published analysis of public data identified five organisational aspects that could explain variability in reliable recovery rates across IAPT sites; early identification of diagnosis to treat, short waiting time between referral and treatment, high average dose of treatment, low percentage of missed appointments and high percentage of people entering treatment [46]. These and other organisational aspects might explain variations in effectiveness of PMHC too. It would therefore be highly useful if the PMHC service adopted a similar monitoring system as the one used by IAPT.

PMHC, like IAPT, can be viewed as a work-in-progress to obtain the goal of “closing the treatment gap” for anxiety and depression in the population. Therefore, it is important to draw on each other’s experiences and also reflect upon which developments to pursue in order to increase the likelihood of reaching this target. Some of the developments seen in IAPT so far are; expanding delivery of NICE recommended treatments for depression, such as interpersonal therapy and brief psychodynamic therapy; national curricula for the training courses that specify key clinical interventions and competencies required to deliver them; regular supervision and implementing the “Plan, Do, Study, Act” methodology to increase recovery rates [35]. IAPT has been estimated to reach out to around 16% of the community prevalence of depression and anxiety disorders and the UK is planning to expand to reach more of the target population [35]. It is less clear how this distribution is in Norwegian context. In light of the mentioned arguments for continuous monitoring of PMHC services, this is an additional reason for keeping track of how much of the target population has been reached.

A related issue is reaching out to underrepresented groups. In the national roll-out of IAPT self-referral was included as a means to include minorities and less represented diagnostic groups such as social phobia [35]. Although referral status was not associated with demographic variables in our sample, this path should remain open to PMHC as it is an important aspect of a low-threshold service. A major planned development in IAPT is to create a new service to reach clients with long term physical health conditions and simultaneously anxiety/depression, as well as clients with medically unexplained symptoms [35]. IAPT has also expanded the service to children and youth [47] and in comparison, PMHC have expanded their target group to include clients from the age of 16.

As shown in this section both PMHC and IAPT are continuing to develop their services. Unfortunately, development does not necessarily equal progress. Further research as well as ongoing monitoring are strongly recommended in order to help identify strengths and weaknesses of the services, evaluate changes, prevent negative developments and increase transparency. In addition, it is also important to document developments of IAPT, PMHC and other descendants of IAPT in academic articles and the like, creating a pool of experiences that other countries can consider and learn from before establishing a service of their own.

### Strengths and limitations

The main strengths of this study where the use of several sources of information and the naturalistic setting. Questionnaires from both therapists and clients have yielded valuable insight into different aspects of implementing PMHC in their municipality. By interviewing the therapists, we gained additional understanding of the implementation processes and discovered possible reasons for deviation from the guidelines.

This study also has a number of limitations. Only two PMHC sites with quite similar community profiles were included in the RCT and this pose a challenge for the generalizability of our findings. However, as noted in the discussion section, our results are in line with the previous Norwegian evaluation of the 12 first pilot sites and, thus, strengthens the potential for generalising to PMHC in Norway. A related issue regarding generalisability is being part of an RCT, and in particular whether monitoring over time may have influenced practice. The therapists themselves reported being more true to the target group and the service model during the data collection period and may therefore have provided an unrealistic good impression of the service. This is a potential threat to all studies where humans are observed, referred to as the Hawthorn effect [48]. According to a systematic review, however, there is no clear empirical evidence for one specific Hawthorn effect [49]. It is acknowledged that there are consequences of research participation, but little is known about the magnitude of the effects and its mechanisms [49]. Nevertheless, because of the relatively long research period, it is conceivable that the therapists got accustomed to the situation and began to work the way they usually do.

The potential of recall bias from the interviewed therapists should also be mentioned as a limitation. The interviews were conducted at the end of the research period and it may be that the therapists did not remember all aspects that could have been relevant for the implementation process.

Finally, it should be mentioned that we did not formally utilize one of the many implementation science frameworks (ISF) [50], but chose a more general approach rooted in basic implementation theory. It is possible that using an ISF would have led to a richer and more complete coverage of the full range of implementation aspects of PMHC. On the other hand, our approach did touch upon domains that are commonly seen in ISF’s. For example, the Consolidated Framework for Implementation Research (CFIR) encompasses the domains intervention characteristics, outer setting, inner setting, individual characteristics, and implementation process. All of these domains were to a greater or lesser extent covered in the present study as well [51].

## Conclusion

This study has provided valuable in-depth information regarding implementation of the Norwegian adaptation of IAPT in two large municipalities; Sandnes and Kristiansand. The results indicated that both sites managed to implement key aspects of PMHC in line with the guidelines provided by NDH, but that further development of the program is warranted. It is recommended to increase the use of evidence-based internet and therapist assisted self-help programmes, to have more specific focus on work participation in treatment, for example with IPS and job specialist or by implementing work-focused CBT, and finally, to establish a continuous monitoring system. Additionally, we want to highlight the benefit of learning from each other’s experiences. As such, we believe that the present paper provides useful insights for other countries considering to adopt the IAPT approach.

## Supplementary information

**Additional file 1.** Interview guide for therapists/service providers at PMHC.

## Data Availability

The datasets generated and/or analyzed during the current study are not publicly available due to ethical restrictions and personal data protection but are available from the corresponding author on reasonable request. The data will be de-identified if made available upon reasonable request.

## Abbreviations

CBT: Cognitive Behavioral Therapy
GAD-7: Generalized Anxiety Disorder scale-7
GP: General Practitioner
NDH: Norwegian Directory of Health
IAPT: Increased Access to Psychological Therapy
IPS: Individual Placement and Support
IQR: Inter Quartile Range
PHQ-9: Patient Health Questionnaire-9
PMHC: Prompt Mental Health Care
RCT: Randomized Control Trial
SD: Standard Deviation
TAU: Treatment As Usual
