# Supplementary material for: Process evaluation of Prompt Mental Health Care (PMHC): the Norwegian version of Improving Access to Psychological Therapies
Source: BMC Health Serv Res. 2020 May 19;20:437. doi: 10.1186/s12913-020-05311-5 (PMC7236093; doi:10.1186/s12913-020-05311-5)
Supplement: Supplementary file 1 — Additional file 1. Interview guide for therapists/service providers at PMHC. [file 12913_2020_5311_MOESM1_ESM.docx]

**Interview guide for therapists/service providers at PMHC**

**Can you start by describing your job role and responsibilities in PMHC?**

1. **How did you get involved with PMHC?**
   1. How did you hear about PMHC?
   2. How and why did you get involved with PMHC?
2. **Where you involved in the design of PMHC in your municipality? If yes:**
   1. How was the service designed? (process & who was involved? How did your municipality decide on the structure and course of your treatment offer)
   2. What were the similarities and differences between the new PMHC service and previous/existing mental health services? (eg no.s of staff; training & qualifications; contractual status; supervision; no of clients seen? Time to first appointment?)
   3. Did the employees of the new PMHC service have the opportunity to complete a training program?
   4. Describe any initial issues or concerns?
3. **How has PMHC run to date?**
   1. What do you think about the PMHC service in your municipality?
   2. What have patient reactions to the PMHC service been like?
   3. Which clients do you see?
      1. Inclusion criteria: to what extent do you follow the inclusion and the exclusion criteria as defined in the RCT study protocol?
      2. Inclusion criteria: What do you think about the in- and exclusion criteria used? (appropriate, too strict or too loose?)
      3. What kind of referral routines have you used during the project period?
      4. How was the balance between prevention and treatment during the project period?
   4. On the PMHC website, it says that you use a stepped care approach
      1. To what extent has this model been followed in practice?
      2. How does your model relate to the “mixed care” model that was often used in the first 12 PMHC pilot sites?
      3. What factors determined the type of treatment clients received?
   5. What routines do you have for using guided self-help and group based psychoeducation (access to standardized self-help packages/group based psychoeducation? sufficiently tried before face-to-face treatment?)
   6. To what extent do you work manual-based in face-to-face treatment? Do you follow manuals for particular disorders? May we get access to these?
   7. How did you design the group-based psychoeducation? (Self-developed? Based on what? Did you get help? How where the courses quality assured?).
   8. How did you design material for the guided self-help? (Self-developed? Based on what? Did you get help? How where the courses quality assured?).
   9. What routines do you have to follow-up client’s work participation (assessing work participation? How? How do you keep a work-focus in treatment? Examples from practice?)
   10. What is your impression of collaborating with general practitioners, primary and secondary care services and the Norwegian Labor and Welfare services?
   11. How have you experienced collaboration with your PMHC colleagues?
   12. To what extent has there been stability in the PMHC team?
   13. To what extent have you experienced that any turmoil in the organization or in the team has affected the service (e.g. work tasks, clients, collaboration with other services and the like?)
   14. To what extent do you think that PMHC has followed guidelines for user involvement and support for children/relatives?
4. **The training program, competence vs tasks**
   1. To what extent has the training from NFKT given you sufficient skills to provide treatment and other work tasks by the NDH guidelines? What was lacking/could be improved?
   2. The guidelines from NDH: clear? To strict?
   3. CBT: “straightjacket” or sensible approach for the target group?
5. **Future developments**
   1. What future changes would you like to see in PMHC if the offer continues?
   2. How should PMHC be continued after the funds from NDH ceases? (have you taken any specific steps to contribute to continuation? Continuation likely? Why?)
6. **The scheme during the data collection period and how the data collection affected routines, environment, meeting with clients.**
   1. How did the routines change during the data collection period? Examples?
   2. Did important changes occur in your routines, arrangements and/or teams during the data collection period?
   3. To what extent has participation in the RCT led to changes in the treatment program? Examples?
   4. To what extent has participation in the RCT affected how clients were met during the data collection period compared to before/after?
   5. How did you experience participating in an RCT?
7. **Any other points/issues not covered in the interview so far? Any points/issues that might be relevant for implementation of PMHC or the results of the RCT study?**
